# Commentary: Extracting robust passage from dynamic change

**DOI:** 10.3389/fpsyg.2023.1123326

**Published:** 2023-08-23

**Authors:** Maria Balcells

**Affiliations:** Department of Philosophy, Bucknell University, Lewisburg, PA, United States

**Keywords:** passage, special theory of relativity, illusory, persistence, motion, time

The passage of time is known by us all too well. We praise it for healing past wounds, lament it for stealing away the warm spring of youth, and curse it for ushering us into the cold dusk of old age. Temporal passage is part of the fabric of our total temporal experience, which we may refer to as manifest time. However, the image of time utilized in science, in particular physics, seems much starker than the image given to us in experience. If we imagine spacetime in physics as being represented by a four-dimensional block containing all events—past, present, and future—then it is hard to see how such a model could adequately capture the passage or flow of time. 

The project of reconciling the tension between these two images requires a multifaceted approach, one that attends not only to the nature of time, but to the nature of ourselves *in* time. Thus, there has been increasing dialogue on the matter between those working in philosophy of time, philosophy of mind, physics, psychology, neuroscience, linguistics, and cognitive science. Gruber et al.'s “*Physical time within human time*” (GBM) and Buonomano and Rovelli's “Bridging the neuroscience and physics of time” (BR) both provide insightful analyses from various disciplines that engage in the project of reconciling the two images (Buonomano and Rovelli, 2021; Gruber et al., 2021). The former does so by utilizing the IGUS^1^ (information gathering and utilizing system) model of the human in time and outfitting it with dualistic components: one that perceives veridically and one that perceives illusorily. The latter does so by examining time as understood by physicists and neuroscientists and proposing a multi-layered understanding of time with the various and seemingly contradictory characteristics manifesting at different levels. Both projects attempt to resolve the tension, in part, by cordoning off those features of manifest time that are either absent from or incompatible with the image of time found in physics. Crucial to the project of bridging the gap between scientific and manifest time is clarifying what exactly is described by the scientific image and what exactly the content of our perceptions is.

Below, I consider how a certain problematic conception of temporal passage, robust passage, often gets injected into our thinking about another less problematic aspect of the manifest image, namely dynamic change. I argue that while robust passage is indeed inconsistent with the scientific image of time, our experience of dynamic change is not. However, they appear in tension with one another because we tend to strip the scientific view of its dynamism and infuse our experiences of dynamic change with beliefs about robust passage.

Robust passage is typically unpacked as something like a unique, moving present that passes over events, bringing them into existence and extinguishing them into the past.^2^ This is the typical target for those who deny the existence of temporal passage on scientific grounds. Einstein's Special Theory undermines the notion of absolute, frame-independent simultaneity and insofar as all events contained in the present occur at the same time, the existence of a unique present is likewise undermined (Putnam, 1967). Without an objectively special present, there is no sense in which the four-dimensional image of time in physics can house robust temporal passage. In fact, the scientific image of time not only lacks robust passage, but is hostile to it. Thus, any experience of robust passage would be illusory.^3^

GBM present a model of an IGUS containing dualistic systems; one veridical and one illusory. They identify three aspects of the manifest image to be incorporated into their model: “(1) a unique (moving) present, (2) dynamism of change/motion, and (3) directionality (temporality)” (GBM, p. 4). The first aspect seems, on the face of it, to be an expression of robust passage and, in fact, GBM point out that an experience of a moving present would be an illusion because of its absence from the scientific image (p. 4). However, GBM further claim that “[t]he actual ‘moving present' is a dynamic illusory experience that is more related if not identical to the experience of “moving”—in other words “motion” (GBM, 4), effectively taking robust passage to be logically equivalent to dynamic motion. In doing so, they preemptively accept that an experience of dynamic change is necessarily illusory as expressed in one of the two principles for their dualistic approach: “[t]he phenomena of dynamism is an experimentally demonstrable illusory experience” (GBM, p. 3).

But, as pointed out by BR, the removal of robust passage from one's model of time does not leave it devoid of change and dynamism. Time itself need not change in order for the four-dimensional image of spacetime to properly represent dynamically changing objects. Time is not an object like bodies, or rivers. Time is the *arena* in which these things move, pass, and flow. To think that time's lack of movement thereby renders the four-dimensional spacetime static is “to imagine an additional external time variable” (BR, p. 4) against which we could judge the static or dynamic character of time. The scientific image of time need only utilize the at-at theory of motion (a term first coined by Russell based on Weirstrauss's development of Analysis in the 19th c.) which defines the motion of an object simply as being “*at* the appropriate point *at* the appropriate time” (Salmon, 1980, p. 137). However, if “[p]hysics is not the description of static entities [but] the description of processes” (BR, p. 4) and physics can adequately describe both static and dynamic processes, then what is the “experimentally demonstrable illusory experience” of dynamism to which GBM refer?

GBM claim that veridical experiences “are congruent with the views of modern spacetime cosmology” (GBM, p. 11) and it would follow that illusory experiences are incongruent with the scientific image or have “no basis in reality” (GBM p. 3). GBM, then, identify veridical and illusory experiences of change as follows: “completed change represents the ‘change' in physics. A dynamic change simply augments that experience” (GBM, p. 6). Their distinction seems to be bound up with the phenomenon of apparent motion and its relation to the experience of real motion. A similar position is discussed by Paul (2010), who argues that because the experience of dynamic motion can be induced even when one is presented only with static images (e.g., the phi-phenomenon) the real motion that we experience in the presence of a continuously moving object is likewise illusory. According to this view, in both cases a kind of dynamism or animation is “painted on” the discrete states.

However, it is important to clarify what the discrete states are in these cases. Mather (2006) explains that the neural substrate for motion processing begins with photoreceptors which are spread across the retina and capable of detecting a change in illumination, for instance, when an object moves across the visual field and obscures or exposes background light, or when an illuminated dot appears and disappears. Paired together, these photoreceptors send discrete signals to a third comparator neuron. If received in the relevant way, the comparator neuron will output a signal that motion has occurred. While this form of motion processing deals with discrete bits of information about change in illumination, *what* that information is about is a change in the world, i.e., a dynamic process. GBM take the position that because the path from the world to the experience requires the brain to fill in some information, it is illusory: “[o]n the one hand “filling in” is illusory, on the other hand the brain guesses correctly” (GBM, p. 3).

However, it is not clear why, even in the case of the brain guessing correctly, we ought to consider the experience illusory. Deeming something to be illusory (or incongruent) only makes sense against an appropriate backdrop of veridicality (or congruence). In the case of apparent motion, it makes sense to say that there is incongruence between the world and the experience because the input involves distinct entities that are spatiotemporally discontinuous and yet we experience a single, continuously moving object. But making the further claim that our experience of dynamic, real motion is incongruent (even if not inconsistent) with the world requires one to treat the world as being composed of discrete, instantaneous events and further, that the collection of those events does not constitute a dynamic process. But if something being dynamic merely means that it changes over time, then this can be described by physics. If it means something more, then it seems that robust passage is being snuck in. Hoerl refers to this position as *error theory*, which requires that “to get the phenomenology of perceptual experiences of movement and change right, we have to introduce the idea that such temporal experiences (at least sometimes) involve the seeming presentation of passage as … a mind-independent change all things in time are subjected to, which consists in their passing from the future, through the present, and into the past” (Hoerl, 2014, p. 2; see also Baron and Miller, 2015). What we have is a mistaken belief that the experience of dynamic change is an experience bound up with robust passage (a belief that stems, in part, from thinking that the way time passes must be akin to how objects change). Therefore, GBM's distinction between illusory and veridical perceptions of change places the mistake at the wrong level of cognition.

On a final note, GBM also consider the experience of dynamic change to be a “double illusion” because it falsely represents the flow of time *and* falsely represents an object as enduring *through* the change (see also Ismael, 2011; Prosser, 2018). They claim that “[p]hysical continuity is not in the cosmological scheme. Instead, what is expected by them is that events…be discrete” (GBM, p. 7). This assumes that all experiences of persistence (or physical continuity) are experiences of endurance, a view that requires the self-same individual to be present at each moment. However, endurance is only one of many philosophical views of persistence (Kurtz, 2006). The views of perdurantism and exdurantism, as well as some other forms of four-dimensionalism, are all perfectly consistent with the scientific image of time and do not require a more robust connection between temporal parts than spatiotemporal continuity which is, at least at some level, described by physics. Further, our experience of an object moving involves the utilization of an object file, “a midlevel visual representation that ‘sticks' to a moving object over time on the basis of spatiotemporal properties and stores (and updates) information about that object's properties” (Noles et al., 2005, p. 325). It is not obvious that the object files represent endurance (a metaphysical view) rather than mere persistence. The more stripped-down version of persistence seems to be assumed even by GBM when they consider the experience of completed change veridical. After all, I experience the hour hand of the clock in a different position now than it was earlier because I represent *the same hour hand* at both times.

If the scientific image includes descriptions of dynamically changing objects, then we ought not think that our experiences of dynamically changing objects are incongruent with the world. Indeed, we may come to have the mistaken belief that those experiences are more robust than they are, but this is a cognitive mistake, not a perceptual one. Further, this mistaken belief taints the way we view the scientific image and causes us to treat it as more impoverished than it truly is. Thus, we should be mindful of what the scientific image expresses. And while there are certainly aspects of the manifest image of time that are in tension with the scientific image, we should treat our experience of dynamic change to be a case where we get things right.

## Author contributions

The author confirms being the sole contributor of this work and has approved it for publication.

## Footnotes

^1^This model of the human was first introduced by Hartle (2005) and later expanded upon by Ismael (2015, 2017) and Callender (2017).

^2^This way of framing robust passage is often attributed to Broad (1923), in particular in his description of passage as representable as a policeman's spotlight passing over houses (read: events).

^3^Some argue that even if there were robust passage in the world, it would not be the kind of thing that could be experienced directly because it is not the kind of thing that is causally efficacious. Thus, it would not cause in us a percept of passage (Price, 2011; Prosser, 2013).
